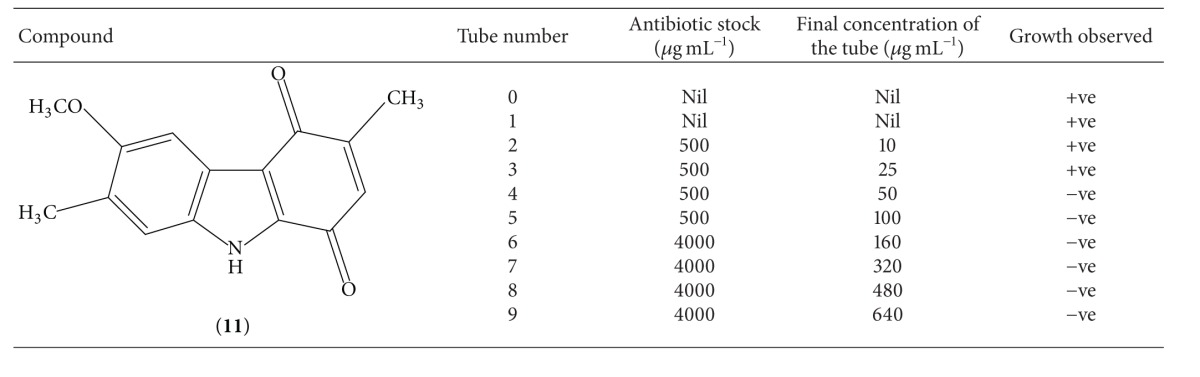# Antibacterial Activity of Murrayaquinone A and 6-Methoxy-3,7-dimethyl-2,3-dihydro-1*H*-carbazole-1,4(9*H*)-dione

**DOI:** 10.1155/2014/540208

**Published:** 2014-05-20

**Authors:** Biswanath Chakraborty, Suchandra Chakraborty, Chandan Saha

**Affiliations:** ^1^Department of Biochemistry & Medical Biotechnology, School of Tropical Medicine, Kolkata 700073, India; ^2^Department of Clinical & Experimental Pharmacology, School of Tropical Medicine, Kolkata 700073, India

## Abstract

The antibacterial activity of Murrayaquinone A (**10**), a naturally occurring carbazoloquinone alkaloid, and 6-methoxy-3,7-dimethyl-2,3-dihydro-1*H*-carbazole-1,4(9*H*)-dione (**11**), a synthetic carbazoloquinone, both obtained during the development of the synthesis of Carbazomycin G, having unique quinone moiety, was studied against Gram-positive (*Bacillus subtilis* and *Staphylococcus aureus*) and Gram-negative (*Escherichia coli* and *Pseudomonas* sp.) bacteria. Compound **10** showed antibacterial activities against both of *Escherichia coli* and *Staphylococcus aureus* whereas compound **11** indicated the activity against *Staphylococcus aureus* only. Both compounds **10** and **11** exhibited minimum inhibitory concentration (MIC) of 50 **μ**g mL^−1^ against *Staphylococcus aureus*.

## 1. Introduction

In 21st century, the most important and challenging problem to the medicinal chemists is to fight against the drug-resistant bacteria. It has been established that the antibacterial resistance is associated with an increase in morbidity and mortality. Frequently, it is recommended to use new antibacterial agents with enhanced broad-spectrum potency. Therefore, recent efforts have been intended for exploring novel antibacterial agents.

After the first isolation of Murrayanine, 3-formyl-1-methoxycarbazole, a carbazole alkaloid having antibiotic properties from* Murraya koenigii* Spreng [1–3], chemists have a significant interest in the field of carbazole alkaloids due to their interesting structural features and potential pharmacological activities [4–7]. Carbazole derivatives having nitrogen containing rigid aromatic heterocyclic moiety with desirable electronic charge transfer properties along with an extended *π*-conjugated system [8] exhibit diverse biological activities such as antibacterial [3, 9, 10], antifungal [11, 12], antiviral [13], anticancer [14], and various other activities. Besides the general antibacterial activity, carbazoles were shown to have a significant antituberculosis activity [15, 16]. This aspect is of interest to the present work, since the highest anti-TB activities were found for carbazole-1,4-quinones.

**Figure 1:**
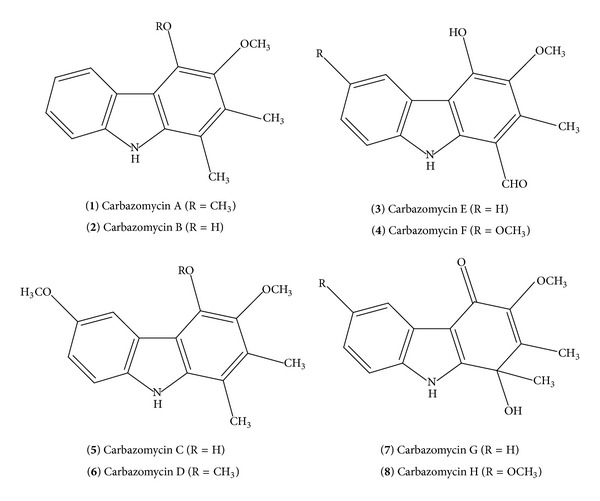
Carbazomycin alkaloids.

The enormous growth of carbazole chemistry has got novel prospect after the isolation of carbazomycins. Carbazomycin alkaloids** 1**–**8** were first isolated by Nakamura and his group from* Streptoverticillium ehimense H* 1051-MY10 [17–23] as shown in Figure 1. In addition, literature survey showed that carbazomycins A, B, C, and D have also been successfully synthesized [24–27].

Carbazomycin A (**1**) and Carbazomycin B (**2**) have been found to be useful antibacterial and antifungal agents and Carbazomycin B was found to be the most potent among the carbazomycins. Both inhibit the growth of phytopathogenic fungi and exhibit antibacterial and antiyeast activities [17]. Carbazomycin B (**2**) and Carbazomycin C (**3**) were shown to inhibit 5-lipoxygenase [28]. Carbazomycin G (**7**) shows antifungal activity against* Trichophyton *species [23]. In addition, extensive photophysical and photochemical properties [29–32] of carbazole nucleus have encouraged the researchers to explore for the synthesis of novel derivatives that have potential biological activities.

**Figure 2:**
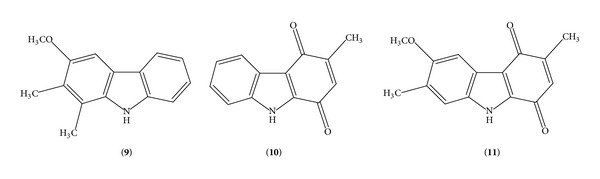
Structure of Deoxycarbazomycin B (**9**), 3-methyl-1*H*-carbazole-1,4(9*H*)-dione, Murrayaquinone A (**10**), and 6-methoxy-3,7-dimethyl-1*H*-carbazole-1,4(9*H*)-dione (**11**).

Synthesis of new molecules which are novel yet resemble well-known biologically active compounds by virtue of their critical structural similarity is the key feature of drug designing program. In this connection it is worthwhile to mention that 4-deoxycarbazomycin B (**9**) (Figure 2), a deoxygenated product of Carbazomycin B (**2**), presented considerable inhibitory activity [33] against various Gram-positive and Gram-negative bacteria. With the advancement in the synthesis of carbazomycin alkaloids, Carbazomycins G and H, which contain a unique quinol moiety, became an attractive synthetic target for several groups due to their challenging congested substitution pattern and their well-known biological activities. It is worth mentioning that first total syntheses of carbazomycins G and H were achieved by Knölker et al. [34–37]. Naturally occurring carbazoloquinone alkaloid, Murrayaquinone A (**10**), containing a quinone moiety having structural similarity with Carbazomycins G and H, has been detected to have a cardiotonic activity on the guinea pig papillary muscle [38]. In addition, during the development of the synthesis of Carbazomycin G [39] in our laboratory by a new synthetic route, a new method is sprung up to obtain carbazoloquinones via cerium-(IV) ammonium nitrate (CAN) mediated oxidation [40] of keto-tetrahydrocarbazoles. Consequently we were able to prepare 6-methoxy-3,7-dimethyl-2,3-dihydro-1*H*-carbazole-1,4(9*H*)-dione (**11**) as well as Murrayaquinone A (**10**) [41, 42]; both the compounds** 10** and** 11** (Figure 2) contain unique quinone moiety having structural resemblance to Carbazomycin G (**7**). This structure-activity relationship boosted us to evaluate the antibacterial activity of these two synthesized compounds against* Escherichia coli*,* Pseudomonas* sp.,* Bacillus subtilis,* and* Staphylococcus aureus* which are commonly used for the antimicrobial studies of carbazole derivatives.

## 2. Materials and Methods

### 2.1. Materials

Nutrient broth, Muller-Hinton broth, and agar powder were purchased from Himedia. Dimethylsulphoxide (DMSO) was purchased from E. Merck. Reference antibiotic disks were purchased from Himedia. The other materials were purchased from E. Merck (India). Compound** 10 **and compound** 11 **used in this work were synthesized in our laboratory.

### 2.2. Bacterial Cultures

Bacterial cultures of* Escherichia coli* (MTCC 42),* Pseudomonas* sp. (MTCC 6199),* Bacillus subtilis* (MTCC 111), and* Staphylococcus aureus* (MTCC 96) were obtained from the Microbial Type Culture Collection (MTCC), Institute of Microbial Technology (IMTECH), Chandigarh, India. These strains were maintained on nutrient agar slants, subcultured regularly (every 30 days), and stored at 4°C as well as at −80°C by preparing suspensions in 10% glycerol.

### 2.3. Synthesis of Murrayaquinone A (**10**) and 6-Methoxy-3,7-dimethyl-2,3-dihydro-1*H*-carbazole-1,4(9*H*)-dione (**11**)

**Scheme 1:**
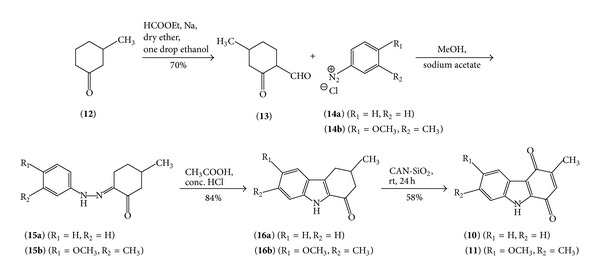
Synthesis of** 10** and** 11**.

A Claisen condensation [43] was carried out on 3-methylcyclohexanone with ethyl formate using metallic sodium in dry ether in presence of one drop of ethanol to furnish 4-methyl-2-oxocyclohexanecarbaldehyde (**13**). This formyl derivative (**13**) on subsequent condensation with proper phenyldiazonium chloride (**14**) under Japp-Klingemann condition [33] yielded 3-methyl-phenylhydrazono-cyclohexanone derivatives (**15**) which on acid catalysed Fischer Indole Cyclisation [33] in concentrated hydrochloric acid and glacial acetic acid mixture afforded ketotetrahydrocarbazole (**16**). Finally, CAN-SiO_2_ mediated oxidation [40] of** 16** at room temperature furnished the expected quinones** 10** and** 11**, respectively (Scheme 1).

*Compound 10.* m.p. 238°C (dec.). IR (KBr, *ν* cm^−1^): 3443, 3217, 1662, 1635. UV *λ*_max⁡_ (MeOH): 222 (sh), 252, 380. ^1^H-NMR (DMSO-d_6_, 500 MHz) *δ* (ppm): 2.41 (s, 3H, C_3_–CH_3_), 6.55 (s, 1H, C_2_–H), 7.19 (s, 1H, C_6_–H), 7.24 (s, 1H, C_7_–H), 7.40 (s, 1H, C_8_–H), 7.80 (s, 1H, C_5_–H), 12.67 (s, 1H, N–H, exch.). ^13^C-NMR (DMSO-d_6_, 125 MHz) *δ* (ppm): 15.57, 113.46, 114.93, 120.89, 123.89, 126.99, 128.01, 131.54, 135.75, 135.84, 147.85, 179.94, 182.33. HRMS *m*/*z*: 212.0705 (Calcd for C_13_H_9_NO_2_H: 212.0708).

*Compound 11.* m.p. 222°C (dec.). IR (KBr, *ν* cm^−1^): 3318, 3024, 1724, 1655, 1616. UV *λ*_max⁡_ (MeOH): 220, 258, 380. ^1^H-NMR (DMSO-d_6_, 500 MHz) *δ* (ppm): 2.03 (s, 3H, Ar–CH_3_), 2.43 (s, 3H, C_3_–CH_3_), 3.81 (s, 3H, Ar–OCH_3_), 6.67 (s, 1H, C_2_–H), 7.47 (s, 1H, C_8_–H), 7.58 (s, 1H, C_5_–H). ^13^C-NMR (DMSO-d_6_, 125 MHz) *δ* (ppm): 15.72, 16.31, 62.58, 113.22, 113.39, 117.54, 131.5, 131.65, 134.68, 137.55, 138.16, 146.08, 148.24, 179.48, 181.28. HRMS *m*/*z*: 278.0794 (Calcd for C_15_H_13_NO_3_Na: 278.0793).

### 2.4. Inoculum Preparation

Inoculums were prepared by transferring three to five well-isolated colonies of identical morphology to 5 mL sterile nutrient broth from the respective nutrient agar plates. The broth cultures were then incubated for 24 h at 37°C. Before the addition of inoculum the turbidity of the actively growing bacterial suspension was adjusted to match the turbidity standard of 0.5 McFarland units prepared by mixing 0.5 mL of 1.75% (w/v) barium chloride dihydrate with 99.5 mL 1% (v/v) sulphuric acid.

### 2.5. Antibacterial Activity Assay

Antibacterial activity was assayed with the standard agar well diffusion method (NCCLS 2000). Muller-Hinton agar plates were surface inoculated uniformly with 100 *μ*L of overnight incubated bacterial suspension (10^6^ CFU/mL) and wells were cut from the agar. Test compounds were dissolved in DMSO and sterilized by filtration through 0.22 mm sterilizing Millipore express filter (Millex-GP, Bedford, OH, USA). Concentrations of the antimicrobial agents used for this assay were 2560 *μ*g mL^−1^, 1280 *μ*g mL^−1^, and 640 *μ*g mL^−1^. 100 *μ*L of these solutions was dispensed into the, respectively, labeled wells. Ciprofloxacin was used as positive reference standard to compare the efficacy of tested compounds and DMSO was used as negative control. The inoculated plates were then kept in the refrigerator for 30 min and then incubated at 37°C for 24 h. After incubation the diameter of zone of inhibition surrounding the wells was measured in millimeters (mm) to evaluate the antibacterial activity of the compounds. Each testing was performed in triplicate. Results were interpreted in terms of diameter (mm) of zone of inhibition.

### 2.6. Minimum Inhibitory Concentration (MIC) Determination

Minimum inhibitory concentration (MIC) of compound is defined as the lowest concentration that will inhibit the visible growth of a microorganism after overnight incubation. MIC values were determined by broth dilution method. The protocol used for this determination is shown in Table 1.

The inoculum was prepared using overnight broth culture of each bacterial strain adjusted to a turbidity equivalent to a 0.5 McFarland standard. The final volume in each tube was adjusted by adding 2.5 mL of sterile nutrient broth.

## 3. Results and Discussions

In the present work the* in vitro* antibacterial activity of Murrayaquinone A (**10**) and 6-methoxy-3,7-dimethyl-2,3-dihydro-1*H*-carbazole-1,4(9*H*)-dione (**11**), obtained via the synthetic route (Scheme 1), was screened against* Escherichia coli*,* Pseudomonas* sp.,* Bacillus subtilis, *and* Staphylococcus aureus*. The results are listed in Table 2. From the data it is clear that 3-methyl-1*H*-carbazole-1,4(9*H*)-dione (Murrayaquinone A,** 10**) possess high activity against both of* Escherichia coli* and* Staphylococcus aureus. *It shows more activity against* Staphylococcus aureus* than* Escherichia coli*. On the other hand, 6-methoxy-3,7-dimethyl-2,3-dihydro-1*H*-carbazole-1,4(9*H*)-dione (**11**) has shown antibacterial activity only against* Staphylococcus aureus.*

As both these compounds have shown sensitivity against* Staphylococcus aureus*, we had performed the experiment for determining the minimum inhibitory concentration of compounds** 10** and** 11** against this organism. Results of this experiment are mentioned in Tables 3 and 4, respectively. Analysis of results shows that both these compounds have MIC value of 50 *μ*g mL^−1^ against* Staphylococcus aureus*.

As per our knowledge, this is the first report where antibacterial activity is detected on carbazoloquinone derivatives. Though the compounds exhibit antibacterial properties, they do not compare very well with generally used standard antibiotics. However, we are expecting that exploring this knowledge with some further structural modifications will yield promising results.

## 4. Conclusions

This report presents the pioneering findings on the potent antibacterial activity of compounds** 10** and** 11** against* Staphylococcus aureus* which has presently acquired resistance against many well-known antibiotics. Again novelty of these synthesized compounds with highly efficient synthetic protocols, along with their pronounced antibacterial activities, largely supports them as potential antibiotics. Further research in this area is likely to yield potent antibacterial compounds against fast-developing and notorious drug resistant bacterial strains.

## Figures and Tables

**Table 1:** Protocol for the determination of minimum inhibitory concentration.

Antibiotic stock (*μ*g mL^−1^)	Vol. of antibiotic (mL)	Vol. of water (mL)	Vol. of inoculum (mL)	Final vol. (mL)	Final concentration (*μ*g mL^−1^)
Nil Nil 500 500 500 500 4000 4000 4000 4000	Nil Nil 0.1 0.25 0.50 1.0 0.2 0.4 0.6 0.8	2.5 2.45 2.35 2.20 1.95 1.45 2.25 2.05 1.85 1.65	0.00 0.05 0.05 0.05 0.05 0.05 0.05 0.05 0.05 0.05	5 5 5 5 5 5 5 5 5 5	Nil Nil 10 25 50 100 160 320 480 640

**Table 2:** Result of antimicrobial activity assay by agar well diffusion method.

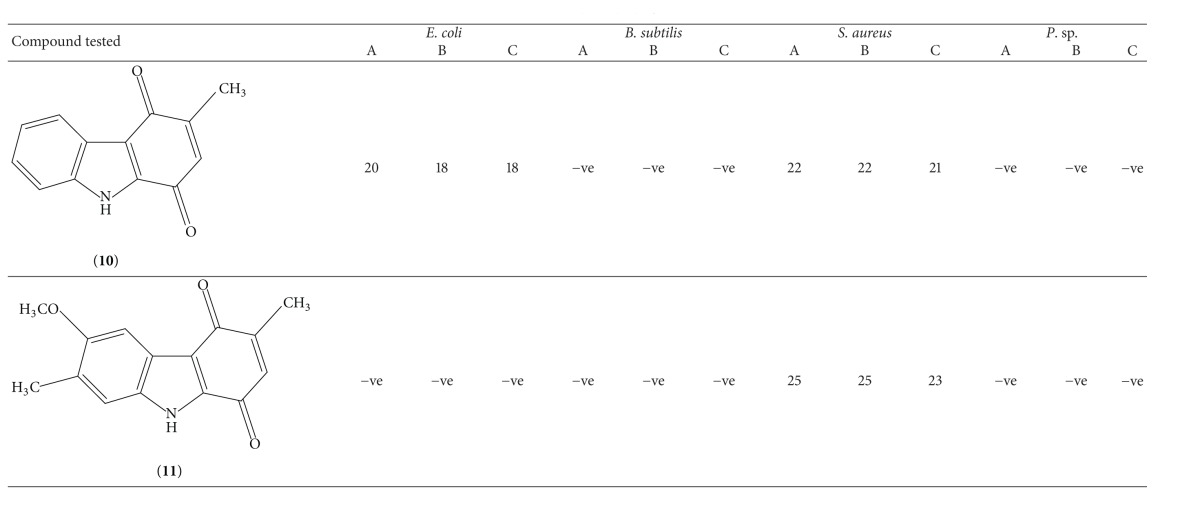

Concentrations: A = 2560 *μ*g mL^−1^; B = 1280 *μ*g mL^−1^; C = 640 *μ*g mL^−1^. Zone of inhibition given in mm (diameter). −ve: no inhibitory activity.

**Table 3:** Result of MIC determination of compound **10**.

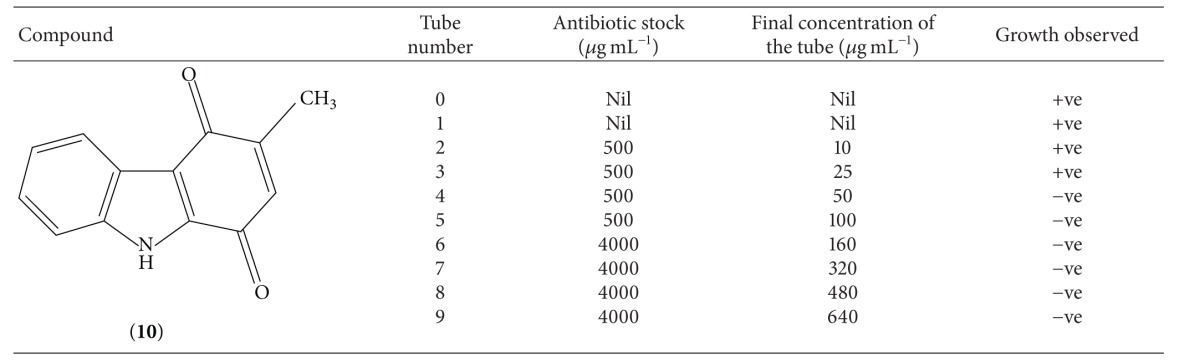

**Table 4:** Result of MIC determination of compound **11**.